# Lamellipodin-Deficient Mice: A Model of Rectal Carcinoma

**DOI:** 10.1371/journal.pone.0152940

**Published:** 2016-04-05

**Authors:** Cassandra L. Miller, Sureshkumar Muthupalani, Zeli Shen, Frauke Drees, Zhongming Ge, Yan Feng, Xiaowei Chen, Guanyu Gong, Karan K. Nagar, Timothy C. Wang, Frank B. Gertler, James G. Fox

**Affiliations:** 1 Division of Comparative Medicine, Massachusetts Institute of Technology, Cambridge, MA, United States of America; 2 David H Koch Institute for Integrative Cancer Research, Massachusetts Institute of Technology, Cambridge, MA, United States of America; 3 Division of Digestive and Liver Diseases, Columbia University, New York, NY, United States of America; 4 Department of Biological Engineering, Massachusetts Institute of Technology, Cambridge, MA, United States of America; Fox Chase Cancer Center, UNITED STATES

## Abstract

During a survey of clinical rectal prolapse (RP) cases in the mouse population at MIT animal research facilities, a high incidence of RP in the lamellipodin knock-out strain, C57BL/6-*Raph1*^*tm1Fbg*^ (Lpd^-/-^) was documented. Upon further investigation, the Lpd^-/-^ colony was found to be infected with multiple endemic enterohepatic *Helicobacter* species (EHS). Lpd^-/-^ mice, a transgenic mouse strain produced at MIT, have not previously shown a distinct immune phenotype and are not highly susceptible to other opportunistic infections. Predominantly male Lpd^-/-^ mice with RP exhibited lesions consistent with invasive rectal carcinoma concomitant to clinically evident RP. Multiple inflammatory cytokines, CD11b+Gr1+ myeloid-derived suppressor cell (MDSC) populations, and epithelial cells positive for a DNA damage biomarker, H2AX, were elevated in affected tissue, supporting their role in the neoplastic process. An evaluation of Lpd^-/-^ mice with RP compared to EHS-infected, but clinically normal (CN) Lpd^-/-^ animals indicated that all of these mice exhibit some degree of lower bowel inflammation; however, mice with prolapses had significantly higher degree of focal lesions at the colo-rectal junction. When *Helicobacter* spp. infections were eliminated in Lpd^-/-^ mice by embryo transfer rederivation, the disease phenotype was abrogated, implicating EHS as a contributing factor in the development of rectal carcinoma. Here we describe lesions in Lpd^-/-^ male mice consistent with a focal inflammation-induced neoplastic transformation and propose this strain as a mouse model of rectal carcinoma.

## Introduction

Rectal prolapse (RP) is a common clinical condition in laboratory mice and is often associated with lower bowel inflammation. More proximal inflammation in the colon can result in thickened edematous tissue and tenesmus. These factors, coupled with the relatively short distal colon that is not rigidly fixed by serosa, provide pathophysiological basis for RP to occur with lower bowel inflammation [1–3]. Bacteria most often associated with this condition are the enterohepatic *Helicobacter* species (EHS) and *Citrobacter rodentium*, although in theory any pathogenic bacteria causing colitis may predispose mice to RP [4–7].

In a recent survey of laboratory mice at our institution, we investigated cases of RP and identified patterns in mouse strain susceptibility, RP association with EHS, and determined the presence of unique histopathological findings [8]. During this study, we identified a transgenic mouse strain, the lamellipodin knockout (Lpd^-/-^), which had the highest incidence of RP of any single strain among the mice housed at MIT, comprising 26% of the mice with RP [8].

Lamellipodin (Lpd) is an Ena/VASP ligand involved in actin dynamics and formation of membrane projections including filopodia and lamellipodia [9]. In response to phosphoinositide signaling via its pleckstrin homology (PH) domain, Lpd plays a key role in fibroblast and melanoblast motility and in axon guidance during neurodevelopment, as well as in breast cancer cell invasion and metastasis [10–14]. Lpd also contains a Ras-association (RA) domain, and although its complete binding properties are not fully understood, Lpd has been linked to both Rac and M-Ras signaling [15, 16]. Further, direct interaction of Lpd with the WAVE regulatory complex can drive activation of Arp2/3. Lpd depletion impairs lamellipodia formation, a phenotype dependent on Arp2/3 activation but not Ena/VASP [9, 13]. When investigating neuronal migration in mouse embryos, absence of Lpd causes tangential rather than radial-glial migration of bipolar pyramidal neurons [11]. These results fueled creation of an Lpd^-/-^ mouse model on a C57BL/6J background, C57BL/6-*Raph1*^*tm1Fbg*^ (Lpd^-/-^), currently established and maintained at MIT. *In vivo* studies of adult mice, however, have not identified a significant neurologic phenotype as expected based on cell culture and *in utero* experiments. Other spontaneous conditions that have occurred in these mice include sporadic cases of dermatitis and malocclusion, both of which occurred at a normal frequency similar to that recorded for the C57BL/6J strain.

Rectal prolapse associated with microbial pathogens may occur both in immune competent as well as in immunocompromised mice [4, 6, 7]. Certain strains of genetically engineered mice, for example IL-10^-/-^ and Rag-deficient mice, are highly susceptible to typhlocolitis with EHS infection and are used to model IBD and colitis-associated carcinoma [2, 7, 17–24]. It is noteworthy that Lpd^-/-^ mice with RP have no known immunodeficiency and do not appear to be susceptible to spontaneous opportunistic infections.

*Helicobacter spp*., in addition to triggering a robust host inflammatory response, are also associated with neoplasia in some hosts. *Helicobacter pylori*, which commonly colonizes the stomach in humans, is the cause of peptic ulcer disease and associated with both gastric adenocarcinoma and MALT lymphoma [25–28]. *H*. *pylori* is classified as a type I carcinogen by the International Agency for Research on Cancer (IARC). Ferrets naturally infected with *H*. *mustelae* exhibit inflammatory and premalignant lesions that can progress to gastric cancer and parallel the gastric disease in humans infected with *H*. *pylori* [29–32]. In mice, the prototype enterohepatic pathogen *H*. *hepaticus* was initially discovered because of its ability to cause hepatocellular carcinoma in A/J mice, resulting in confounded carcinogenicity studies conducted at the National Cancer Institute [33, 34]. Since then, *H*. *hepaticus* and other EHS including *H*. *typhlonius*, *H*. *rodentium*, and *H*. *bilis*, known to persistently colonize the intestinal crypt of the lower bowel, have been shown to induce colitis-associated cancer in susceptible immunodeficient strains of mice [22, 35–41].

We report on a total of 19 cases of RP in Lpd^-/-^ mice infected with EHS. Of note, only homozygous null mice were affected, and 17 of the 19 (89%) were male. The rectal and immediate adjacent lesions are characterized as an inflammation-associated neoplastic transformation and may prove useful in experimental studies exploring the pathogenesis of rectal carcinoma.

## Methods

### Animals

The Massachusetts Institute of Technology (MIT) Committee on Animal Care approved the use of mice in this study (Animal Welfare Assurance #A3125-01). Over a 1-year period, mice housed at MIT with RP were initially identified via our laboratory animal health monitoring system. Excluded pathogens include most known parasitic, viral, and bacterial mouse pathogens (as determined by our murine sentinel program), although mouse norovirus is not routinely surveyed. With the exception of one barrier facility, mice are not maintained *Helicobacter*-free. An unusually large percentage of RP cases (26%) occurred in Lpd^-/-^ mice. The Lpd animals incorporated into this report can be broadly divided into three categories: 1) A colony of Lpd ^-/-^ mice with clinical findings of RP that were identified over the above 1-year period by qualified animal health care personnel from the institute’s Division of Comparative Medicine (DCM), 2) Lpd (+/+, +/-, and -/-) mice from the same cohort as above with no clinical incidence of RP (CN), 3) Embryo transfer (ET) re-derived Lpd (+/+, +/-, and -/-) mice that were negative for *Helicobacter spp* and maintained for 15 months for RP surveillance and histologic evaluation. All mice were genotyped by ear notching at weaning by a commercial vendor (Transnetyx, Cordova, TN) using real time PCR with specific probes designed for wild type and excised Lpd gene sequences.

### Necropsy

Mice were euthanized via carbon dioxide overdose and submitted for a complete necropsy and specimen processing. Liver and gastrointestinal tract samples were collected and stored at -20°C (for PCR assays), -80°C (cultures and cytokine assays), or in formalin. Parasitological testing included Fecasol^®^ purified sodium nitrate floatation (Vétoquinol USA, Inc., Ft. Worth, TX), anal tape tests, and direct smears of cecal contents. For the Lpd^-/-^ strain, similar processing of tissues was undertaken in CN (without RP) EHS-positive, and EHS-free (rederived) mice. For all animals, histological submissions included liver, entire GI tract from stomach to rectum, and mesenteric lymph nodes. In addition, for a subset (n = 6) of aged matched CN control animals from the Lpd^-/-^ colony with endemic EHS, a systematic gross and histopathological evaluation was performed on multiple tissues including the entire GI tract, liver, spleen, pancreas, kidney, adrenal gland, heart, lungs, thymus, brain, and reproductive organs to identify any underlying genotype-induced pathology. Lpd^-/-^ mice were also grouped to compare differences in pathology by age (2–3 months, 5–6 months, and 8–11 months).

### Helicobacter PCR & RFLP Analysis

DNA was extracted from tissue samples (colonic, cecal, and rectal) by using the High Pure PCR Template Preparation Kit (Roche diagnostics GmbH, Mannheim, Germany) according to manufacturer instructions. Genus-level *Helicobacter spp*. primers (forward C05, reverse C97) [42] were used to amplify a 1.2-kb PCR product, detected by electrophoresis (on 2% agarose gel) and ethidium bromide staining, followed by visualization with UV light. Positive samples were analyzed by restriction fragment length polymorphism (RFLP) as previously described using the HhaI and AluI restriction enzymes[43]. RFLP patterns were confirmed using PCR assays with species-specific primers for *Helicobacter hepaticus* [44] and *Helicobacter typhlonius* [45], with positive controls consisting of DNA extracted from pure cultures of each *Helicobacter* species.

Real-time quantitative PCR of *Helicobacter spp*. was performed on samples from the cecum, colon, and rectum using previously published methods [46]. A standard curve was generated with serial 10-fold dilutions of an *H*. *pylori* SS1 sample. The 16S rRNA gene primers and probe have been previously described [47]. Copy numbers of *Helicobacter spp*. were standardized to murine chromosomal DNA using qPCR with an 18S rRNA gene primer and probe mixture (Applied Biosystems, Foster City, CA) as previously described [46, 48].

### Histopathology

GI and liver tissues were formalin-fixed, processed, and embedded in paraffin; 5μ sections were stained with hematoxylin and eosin. Slides were evaluated in a blinded fashion by a board-certified veterinary pathologist. Lesions in different segments of the large intestine were scored according to an ascending scale from 0 to 4 based on severity for various parameters including inflammation, epithelial defects, edema, crypt atrophy, hyperplasia, and dysplasia/neoplasia [49]. In order to specifically delineate the extent of inflammation in different segments of the intestine, pathological assessments were done at four separate locations (cecum with ileocecocolic junction, proximal and transverse colon, distal colon, and rectum). Criteria for malignancy were determined using guidelines recommended by the Mouse Models of Human Cancers Consortium’s consensus report on colorectal neoplasia [50].

### Immunohistochemistry

Paraffin sections of ceca and colons (including rectums) from EHS-positive RP mice, EHS-positive control mice, and EHS-negative control mice were stained using a standard immunohistochemistry protocol. Briefly, slides were deparaffinized and rehydrated, and then heated to 95°C for 20 minutes for antigen retrieval. Slides were stained using avidin biotin-peroxidase complex kits (Vector Laboratories, Burlingame, CA) and counterstained with hematoxylin. Stains included macrophages (rat-anti-mouse F4/80 antibody, Caltag Laboratories, Burlingame, CA), B lymphocytes (rat anti-mouse CD45R/B220 monoclonal antibody, BD Biosciences, San Jose, CA), T lymphocytes (rabbit-anti-human CD3 antibody, DAKO Cytomation, Carpinteria, CA), and Ki-67 antigen (anti Ki-67, BD Biosciences, San Jose, CA) for qualitative assessment of epithelial cell proliferation.

### Immunostaining and morphometric analysis of DNA damage marker (H2AX)

The presence of DNA damage was studied using a monoclonal antibody against histone H2AX (9718P, Cell signaling, Beverly, MA, USA). Briefly, formalin-fixed paraffin embedded (FFPE) tissue sections were deparaffinized and washed in gradient ethanol 100%, 90%, 70% and water. Antigen retrieval was achieved using Dako modified citrate-based buffer (S1700, Dako, Carpinteria, CA, USA) and boiling at 95°C for 20 minutes. Tissue sections were blocked using 3% BSA, treated with primary antibody (1/200 dilution, 1 hour at room temperature), washed, and treated with secondary antibody (Invitrogen, Alexa-Flour-568 conjugated, 1/500 dilution, 1 hour at room temperature). Tissue sections were counter-stained with DAPI. Images were taken using QIClick digital CCD Camera (QImaging, Surrey, B.C, Canada) mounted on a Zeiss Axioskop 2 plus microscope and the Image Pro-Plus (7.2 version, Media Cybematics, Sliver Spring, MD, USA). Morphometric analysis was performed using Image Pro-Plus 7.2 (Media Cybernetics, Inc., Rockville, MD).

### Fluorescent In Situ Hybridization

Paraffin sections of ceca and colons (including rectums) from EHS-positive RP mice and EHS-positive CN mice were deparaffinized and rehydrated. A combination of two genus-specific probes, HEL274 and HEL717, labeled with Cy3 were used (Integrated DNA Technologies, Coralville, IA) [51]. Hybridization buffer (0.9 M NaCl, 100 mM Tris—HCl, 0.1% SDS, 30% formamide) with 5 ng/mL of each probe was preheated for 10 min at 74.5°C; 80 uL of this solution was added to each slide. Slides were covered in parafilm, and placed in a dark humidification chamber overnight at 48°C. After incubation, slides were rinsed in double-distilled water and serially washed in pre-warmed rinsing buffers for 15 min each (Buffer 1: 0.9 M NaCl, 100 mM Tris—HCl, 0.01% SDS. Buffer 2: 0.9 M NaCl, 100 mM Tris—HCl.). Slides were air-dried, mounted in Vectashield with DAPI (Vector Laboratories, Burlingame, CA), and examined under a Zeiss Axioskop 2 fluorescent microscope. Tissues were considered positive for *Helicobacter spp*. if fluorescent spiral organisms were observed under the rhod filter.

### Quantification of Tissue Inflammatory Mediators

Tissue samples from both the prolapsed portion of the rectum and the proximal colon were collected from mice with RP for cytokine analysis. Total RNA was extracted from samples with Trizol reagent according to manufacturer instructions (Invitrogen, Grand Island, NY). cDNA was generated using the High Capacity complementary DNA Archive Kit (Applied Biosystems, Foster City, CA). Quantitative PCR was performed with the 7500 Fast Real-Time PCR System (Applied Biosystems) for Tnf-α, iNos, Inf-γ, Il-10, Il-1β, Il-4, Il-17, and Foxp3; all data were normalized to endogenous control mRNA expression (glyceraldehyde-3-phosphate dehydrogenase, GAPDH). Rectal tissues from RP mice were compared to both rectal tissues of EHS-negative control mice and to proximal colon tissues of RP animals. EHS-infected colon samples were also available for comparison to proximal colon in diseased vs CN animals. Data were expressed as fold change in reference to control mice.

### Measurement of tissue infiltrating immature myeloid cells (IMC)

Blood and spleen samples from RP mice or controls were lysed by red blood cell (RBC) lysis solution (Biolegend, San Diego, CA). Cells were then passed through 70 um cell strainer (BD Biosciences, Franklin Lakes, NJ) to prepare monolayer cell suspension. Prolapsed or normal rectal tissues were enzymatically digested with enzyme cocktail containing collagenase IV (Worthington Biochemical Corp, Lakewood, NJ), dispase (Gibco), and DNAse I (Roche). Digested cell suspensions were subjected to 40% versus 80% percoll gradient centrifugation (GE Healthcare, Pittsburgh, PA) to enrich leukocytes. To phenotypically analyze immature myeloid cells, cells from blood, spleen, or RP tissue were stained with the following antibodies: PEcy7-CD45 (clone 30-F11), APC-CD11b (clone M1/70), and Percp-Cy5.5-Gr1 (clone RB6-8C5). Multicolor flow cytometry was performed on LSRII flow cytometer (BD Biosciences). All flow antibodies were obtained from Biolegend. Data were analyzed and presented by using Flowjo 9 software (Tree Star, Ashland, OR).

### Statistical Analysis

The data on inflammation-associated gene expression, H2AX DNA damage marker, and CD45+CD11bGr1 myeloid cells were statistically analyzed between the groups using unpaired two-tailed student t tests. Data analysis for histological scores among the groups were compared using the Mann-Whitney nonparametric test. Results were considered significant at p<0.05. Histopathological scores were compared across groups by using the Kruskal-Wallis One-way Analysis of Variance (ANOVA) with Dunn's post-test and between groups by the Mann-Whitney U-test using Prism software (GraphPad, San Diego, CA).

## Results

### Animals

The first part of the study involved a one year screening period for the identification and documentation of the overall incidence of RP in mice maintained at MIT in different experimental and housing conditions [8]. Of particular note was the finding of 19 Lpd^-/-^ mice diagnosed with progressively worsening RPs that had to be euthanized. Strikingly, RP Lpd^-/-^ mice were over-represented and accounted for 26% of all RP cases during the one year observation period. As the other strains of mice with RP are not the main focus of this study, their pathological findings are not detailed but they do represent a broad range of genotypes in different experimental contexts and most of the mice had either severe inflammatory or neoplastic processes affecting multiple segments of colon, cecum as well as other organs in some instances. The RPs in these mice were clinically considered to be secondary to various underlying GI pathologies and/or experimental manipulations. Within the Lpd colony, RP prevalence was estimated to be 1.5%, based on the average number of cases and the Lpd mouse population at various times. The prevalence of RP was not restricted to all mice in a given cage. RP mice often were in cages with CN mice. However, because all RPs occurred in homozygous null mice, the prevalence among this Lpd^-/-^ mouse population was much higher, approximately 5.8%.

Overall, Lpd^-/-^ mice with RP ranged in age from 4 months to 13 months; 17 of 19 were male, and all were homozygous null for the Lpd gene. Control groups included both sexes, mice ranging from 7 weeks to 1 year old, and a mix of wild type, heterozygous, and homozygous mice. Prior to euthanasia, no mice exhibited diarrhea and all appeared in good body condition. All RP mice examined were negative for pinworms by fecal flotation, anal tape test, and direct smears. Other than rectal prolapse, no gross abnormalities were seen in any RP or control mice. We also systematically evaluated 27 clinically normal EHS+ Lpd mice (EHS+), and 58 *Helicobacter*-free (EHS-) Lpd mice with no visible signs of RP for any underlying pathology and colonization parameters. These control mice included an equal distribution of sexes and wild type, heterozygous, and homozygous knock-out genotypes.

### *Helicobacter* spp. prevalence in Lpd^-/-^ mice

**Fig 1:**
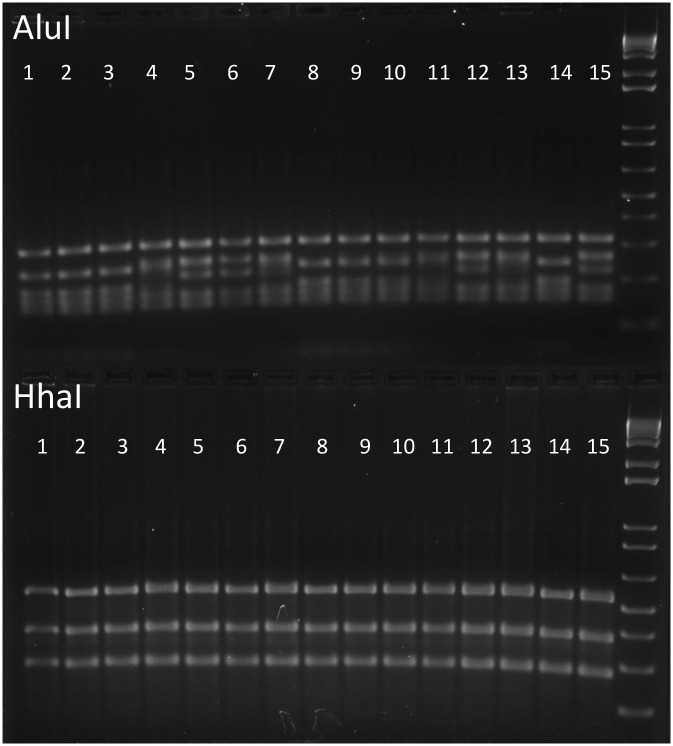
RFLP Patterns for EHS+ Lpd^-/-^ Mice. Results from 15 animals are shown, with AluI digest on the top, and the corresponding HhaI digest shown on the bottom for each sample. While HhaI digests all exhibit the typical banding pattern for *H*. *typhlonius*, there is variability in the AluI patterns. Rows 1, 2, 3, 8, 9, 10, and 14 are all characteristic of *H*. *typhlonius*, whereas the remaining samples showed patterns that were less readily identifiable, suggesting coinfections. These animals were later determined to be co-infected with *H*. *hepaticus* by species-specific PCR.

Genus-specific 16S rRNA PCR was positive for *Helicobacter spp*. for all RP and most CN colony animals. RFLP analysis often showed a banding pattern consistent with *H*. *typhlonius*, although some samples had additional bands that suggested co-infection with *H*. *hepaticus* (Fig 1). Species-specific PCRs confirmed that all RP animals were colonized by *H*. *typhlonius* and 53% were colonized by *H*. *hepaticus*. Of the clinically normal mice, 95% of mice older than 3 months were co-infected with both species; one 8-month-old animal was only infected with *H*. *typhlonius*. Some animals that were younger than 3 months of age were either uninfected or only infected with one *Helicobacter sp*.; this is presumably because an additional specified *Helicobacter sp*. had not colonized these younger mice. All rederived *Helicobacter* spp. free mice were confirmed to be free of EHS by genus-specific fecal PCR.

In addition to the high prevalence of *H*. *hepaticus* and *H*. *typhlonius* in the colony, three of the clinically normal mice were infected with *H*. *mastomyrinus* and one mouse with an unnamed isolate MIT 01–6451, identified by sequencing of the 1.2 kb segment of 16S rRNA. These organisms were not found in the RP mice.

### Colonization density of *Helicobacter* spp. in the lower bowel of Lpd^-/-^ mice

**Fig 2:**
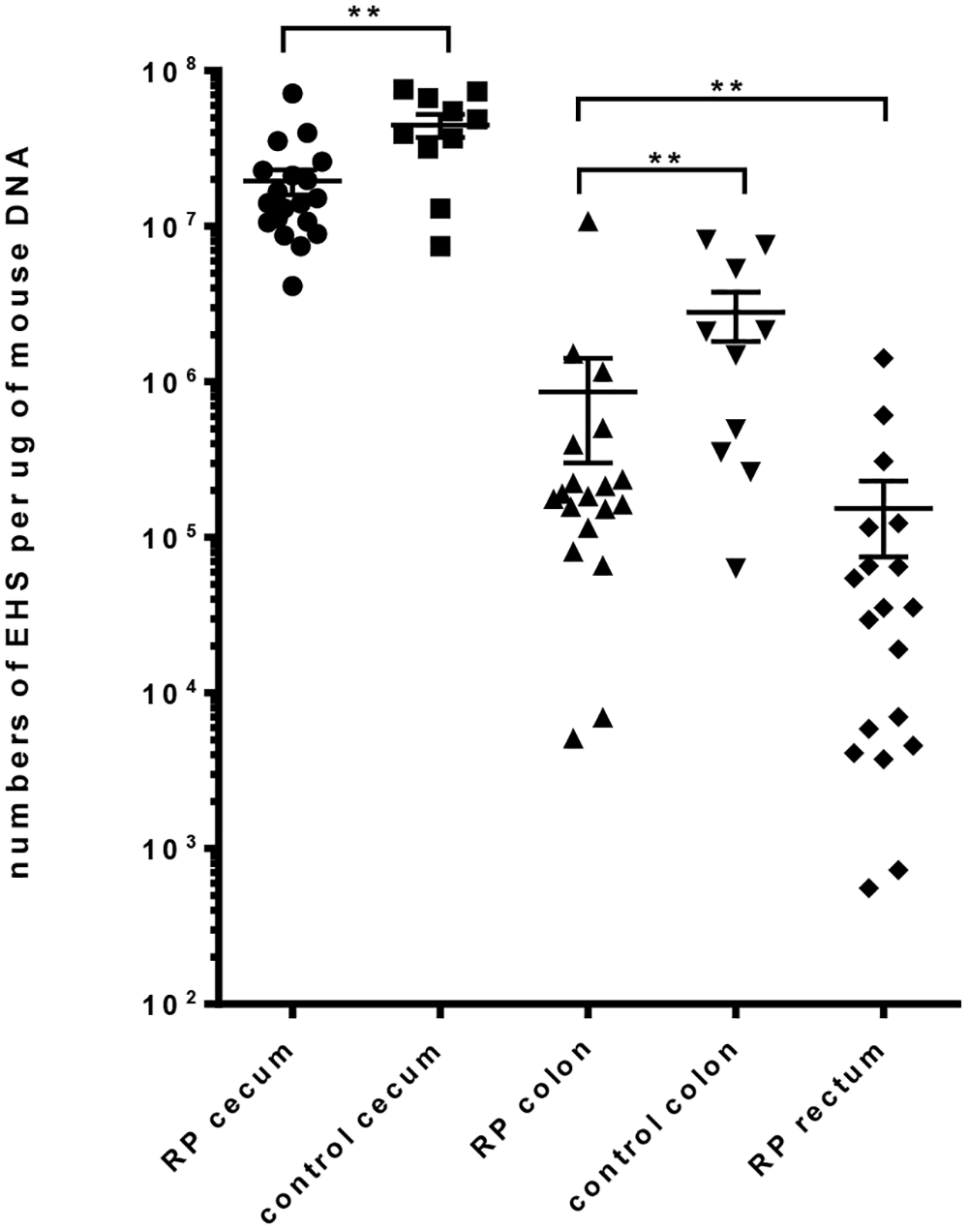
Quantitation of EHS in the Lower Bowel of LPD -/- mice. Levels of EHS colonization were determined by qPCR in the cecum and colon of both RP and CN mice. Organisms were quantified in the rectum only for RP mice, as the normal rectal tissue was less abundant and thus spared for histologic analysis. As expected, EHS were most abundant in the ceca for both groups of mice. Colonization was more variable and tended to decrease distally, yet EHS were still detected in all locations. (RP: Rectal prolapse; CN: Clinically normal).

For quantitative assessment of *Helicobacter* spp. infection, genus-specific qPCR was performed on RP and EHS+ ceca and colons, and on rectums of RP mice (Fig 2). For both groups, ceca were more heavily colonized than colons. Rectums of RP mice had the lowest colonization and a broader range of values, with detectable levels ranging from 10^2^ to 10^5^ bacteria per ug of mouse DNA.

### Histopathological findings in Lpd^-/-^ mice with or without RP

**Fig 3:**
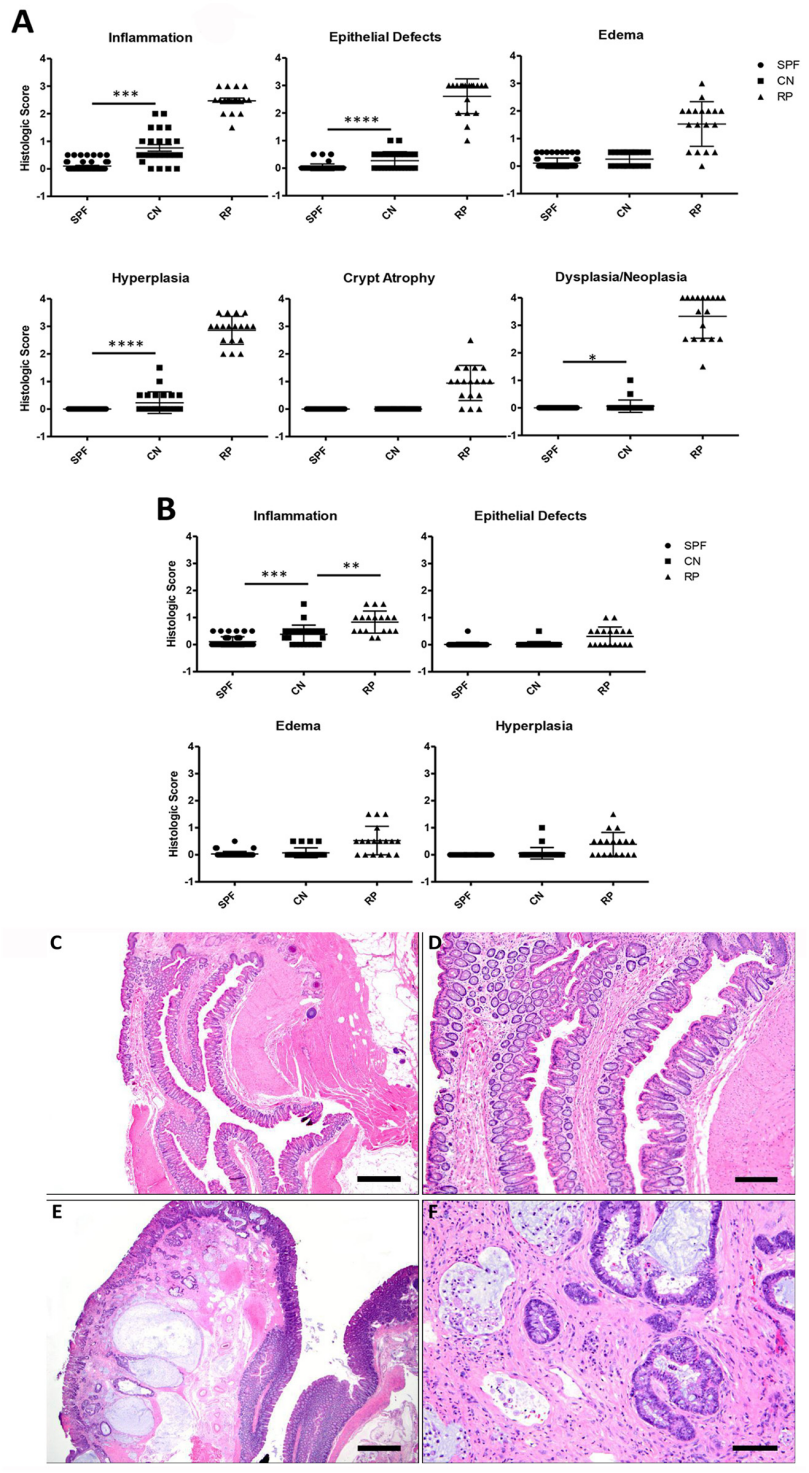
(A) Comparison of Rectal/Colo-rectal Junction Histologic Scores. (B) Comparison of Distal Colon Histologic Scores. (C-F) Comparison of Histology Observed in Clinically Normal Lpd Mice (C&D) vs RP Lpd Mice (E&F). In the low and high magnification images of a representative clinically normal animal, the non- prolapsed rectum and colon are normal without any significant inflammation or other changes like hyperplasia or dysplasia. The low magnification image (E) of the clinically affected animal shows marked expansion of the prolapsed rectum and adjacent distal colon by inflammation, edema, glandular hyperplasia, and high grade dysplasia/neoplasia. High magnification of the prolapsed rectum (F) showing carcinomatous glands that have invaded deep into the underlying musculature. (Bar lengths = C: 400μm; D: 150μm; E: 400μm; F: 75μm).

Qualitative histological assessments and semi-quantitative histological scoring of different segments of the lower bowel in the three groups of Lpd mice showed significant patho-morphological alterations in the prolapsed clinical cases as compared to the CN EHS+ mice and re-derived EHS- mice. In prolapsed cases, the lesions were most severe in the rectum including recto-anal transitional zone with some extent albeit to a lesser degree into the immediate adjacent junctional distal colon. In most cases of rectal prolapse, the prolapsed rectum was characterized by moderate to severe mixed inflammation consisting of neutrophils, macrophages, lymphocytes and eosinophils, as well as epithelial erosions/ulcerations, prominent glandular hyper-proliferation and severe dysplasia/neoplasia. The dysplastic/neoplastic lesions ranged from moderate to high grade dysplasia, intra-epithelial neoplasia, and well-differentiated invasive carcinoma. As frequently seen in mice, morphological appearance of lesions in chronic RP cases can frequently mimic invasive carcinomas. We carefully evaluated the clinical data on the duration of rectal prolapse and histological features such as lack of significant colitis/typhlitis, presence of many listed histopathologic features in the consensus criteria as established by Biovin et.al, to distinguish pseudo-invasion or herniation from true dysplasia with glandular invasion as noted in carcinomas [50]. In cases of RP, frequently the rectum/rectal-colonic junction was markedly thickened and proliferative, forming moderate well differentiated micro-adenomatous to sessile proliferations with significant architectural and/or cytological atypia, visible mitosis, glandular herniation and invasion. The invasive type of moderate to high dysplastic glands were identified by their horizontal spreading or frond like leading edge, in the submucosa and/or submucosa. The invasive glands frequently were cystic with indistinct basement membrane, and often partially lined by epithelium that was filled with mucous and denuded ghost cells. In some instances, individual cells or budding cluster of cells were seen in the frequently thickened stroma. Select representative photographs of lesions are shown in Fig 3. The distal colon and the ano-rectal transition (often not a distinct identifiable region) also had profound increases in inflammation, hyperplasia, epithelial defects, and edema relative to both CN EHS+ and EHS- mice. There was no florid typhlocolitis or colitis typical of EHS-associated disease, previously noted in immunocompromised mouse strains, for either the RP or EHS+ groups. Also absent were hepatomas and hepatocellular carcinomas, seen with *H*. *hepaticus* infection in susceptible mouse strains [52]. The EHS- mice exhibited only low-grade background inflammation throughout the lower bowel, which was more prominent in the cecum than in the colon and rectum.

A complete systemic examination of CN Lpd^-/-^ mice from the colony with enzootic *Helicobacter spp*. infection was performed, with no extra-intestinal lesions being identified. Four lower bowel locations were compared between the three groups. For both EHS+ and EHS- control mice, a range of ages, both sexes, and both heterozygous and homozygous mice were studied. No differences in histology scores were noted based on age, sex, or genotype within these groups. For many of the criteria, all or most animals within a group had scores of zero and these scores did not vary between groups. Those criteria and locations that either showed significant differences between groups or had the majority of scores greater than zero are shown in Fig 3. In the cecum, scores for epithelial defects and edema were slightly elevated in EHS- mice compared to mice with RP. However, all other significant score differences were limited to the distal colon and rectum, with RP mice having higher scores for all parameters shown. The only dysplastic or neoplastic lesions observed were limited to the rectum and colo-rectal transition zone; all RP mice had some degree of dysplasia or neoplasia, typically with scores of 3 to 4. Two mice in the EHS+ group also had dysplastic rectal lesions, but to a lesser degree (scores of 1–2).

Typical morphologic diagnoses for RP in Lpd^-/-^ mice included high grade dysplasia, carcinoma in situ, and well-differentiated invasive carcinoma, frequently accompanied with mucous-filled cystic glands and invasive herniation into the submucosa and muscularis. A total of 58% of RP cases were classified as invasive neoplastic lesions, with an additional 11% being non-invasive tumors. The very distal portion of the colon and the ano-rectal transition (often not a distinct identifiable region) also had profound increases in inflammation, hyperplasia, epithelial defects, and edema relative to both CN EHS+ and EHS- mice. There was no florid typhlocolitis typical of EHS-associated disease seen in immunocompromised mouse strains, for either the RP or EHS+ groups. Also absent were hepatic lesions (hepatitis and hepatocellular carcinoma) sometimes seen with *H*. *hepaticus* infection in susceptible mouse strains. The EHS- mice exhibited only low-grade background inflammation throughout the lower bowel, which was more prominent in the cecum than in the colon and rectum.

### Fluorescent In Situ Hybridization of *Helicobacter* spp.

**Fig 4:**
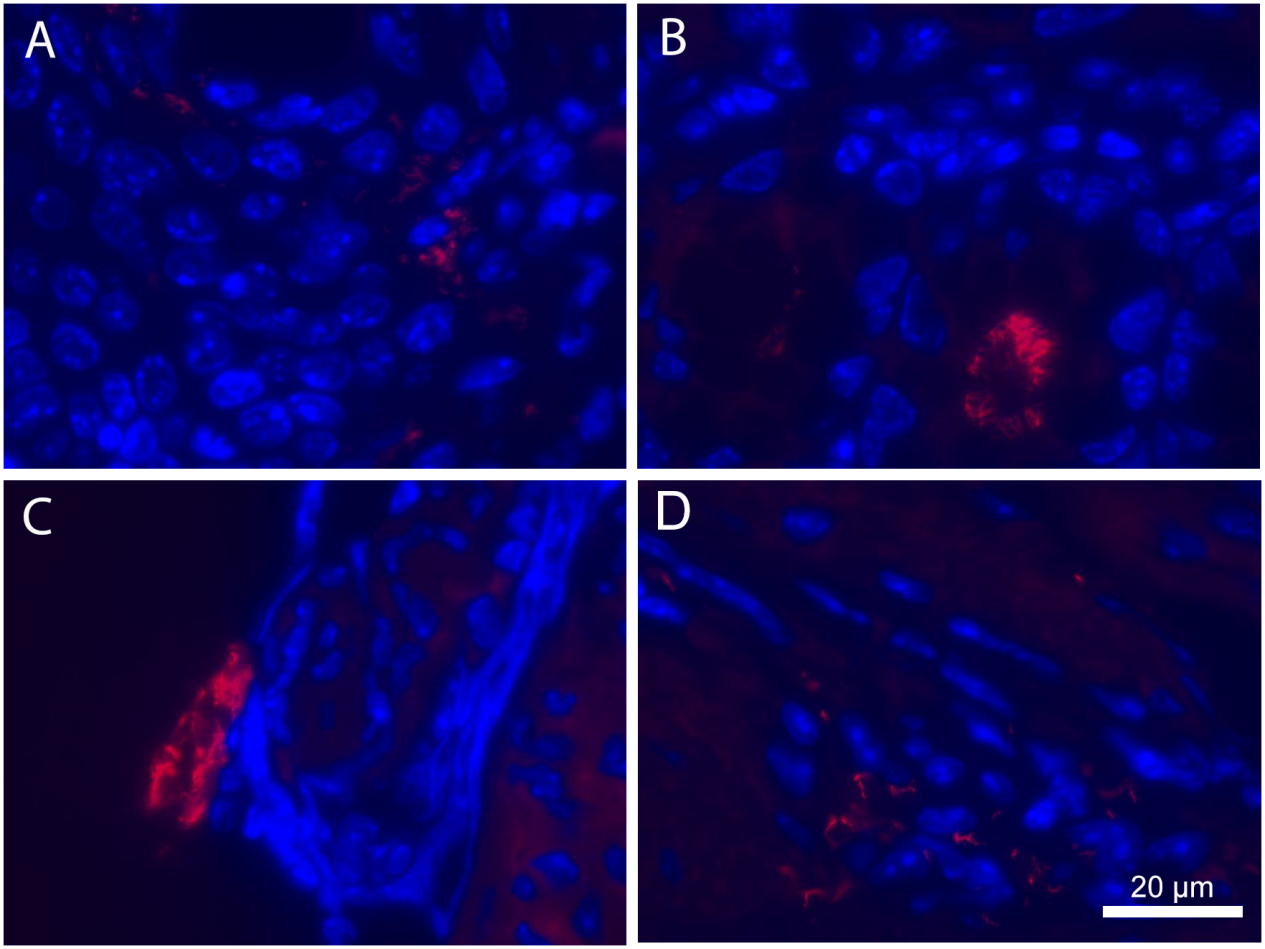
Fluorescent in situ Hybridization on lower bowel of Lpd^-/-^ mice. (A) The colo-rectal junction region of a clinically normal, EHS-positive mouse showing organisms adjacent to host cells in the distal GI tract; (B) Typical positive staining showing heavy colonization within the crypts of the colon; (C) Rectum of the same animal in B with organisms detected along the surface of abnormal prolapsed tissue; (D) Another prolapsed rectum with EHS dispersed within the abnormal edematous tissue. A combination of two genus-specific probes, HEL274 and HEL717, labeled with Cy3 were used. Magnification = 630x; bar = 20μm.

Spiral bacteria were easily detected in ceca and colons of both RP and CN EHS+ mice (Fig 4). Large pools of positive-staining spiral bacteria were visible in the lumen of the proximal colon in all specimens examined. This region of the colon also had prominent bacterial cells frequently detected along the apical surfaces within the crypts. Moving distally in the colon, there was a marked decrease in bacteria detected, but organisms were still present in all specimens examined. *Helicobacter* spp. were observed within the rectum, occasionally in crypts, and often along the periphery of the prolapse or in association with tissue that had lost normal crypt architecture.

### DNA damage

**Fig 5:**
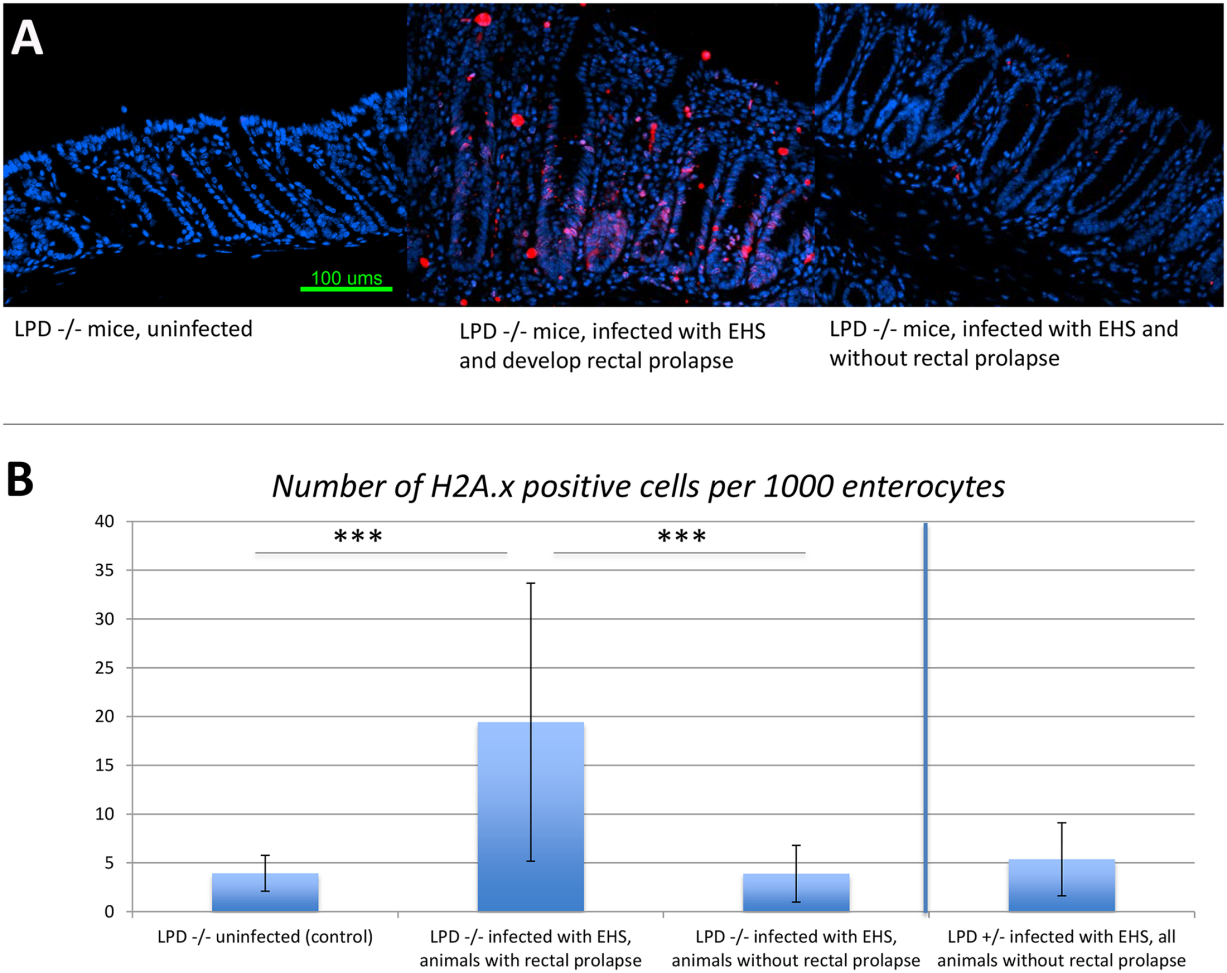
Immunostaining and morphologic analysis of DNA damage using H2AX. (A) immunostaining of DNA damage marker H2AX in LPD -/- mice without EHS infection, LPD -/- mice with EHS infection and with rectal inflammation and prolapse, LPD -/- mice with EHS infection and without rectal inflammation and prolapse; (B) morphometric quantification of average frequency of H2AX positive cells per thousand enterocytes. For each animal, total 8–10 images were obtained and analyzed. Monoclonal rabbit anti-H2AX antibody was used to label cells bearing DNA damage. The secondary antibody was goat anti-Rabbit Ig(H+L), alexa fluor 568 conjugated. Animal number, group LPD -/- control uninfected mice = 6, group LPD -/- EHS-infected with RP = 17, group LPD -/- EHS-infected without RP = 14, group LPD +/- EHS-infected = 10. *** significant at < 0.001. Magnification = 200x; bar = 20μm.

Consistent with the pathological observations, as shown in Fig 5, immunostaining of DNA damage marker H2AX revealed that the Lpd +/- mice with EHS infection, and normal histological appearance, featured little DNA damage (in average 5 positive cells per 1000 enterocytes, non-significant) compared to the rederived uninfected control Lpd^-/-^ mice. (in average 4/1000 enterocytes). This data suggested a protective role of Lpd against infection and inflammation. In contrast, the Lpd^-/-^ mice, with rectal prolapse (Fig 5) with EHS infection, showed significantly increased percentage of H2AX-positive cells (20/1000 enterocytes, p<0.001) compared to the control mice. However, there is a group of animals, belonging to the same Lpd^-/-^ group, which remained healthy even with EHS infection, with no histopathological changes and no changes in the percentage of DNA damage cells (4/1000 enterocytes).

### Quantification of Tissue Inflammatory Mediators

**Fig 6:**
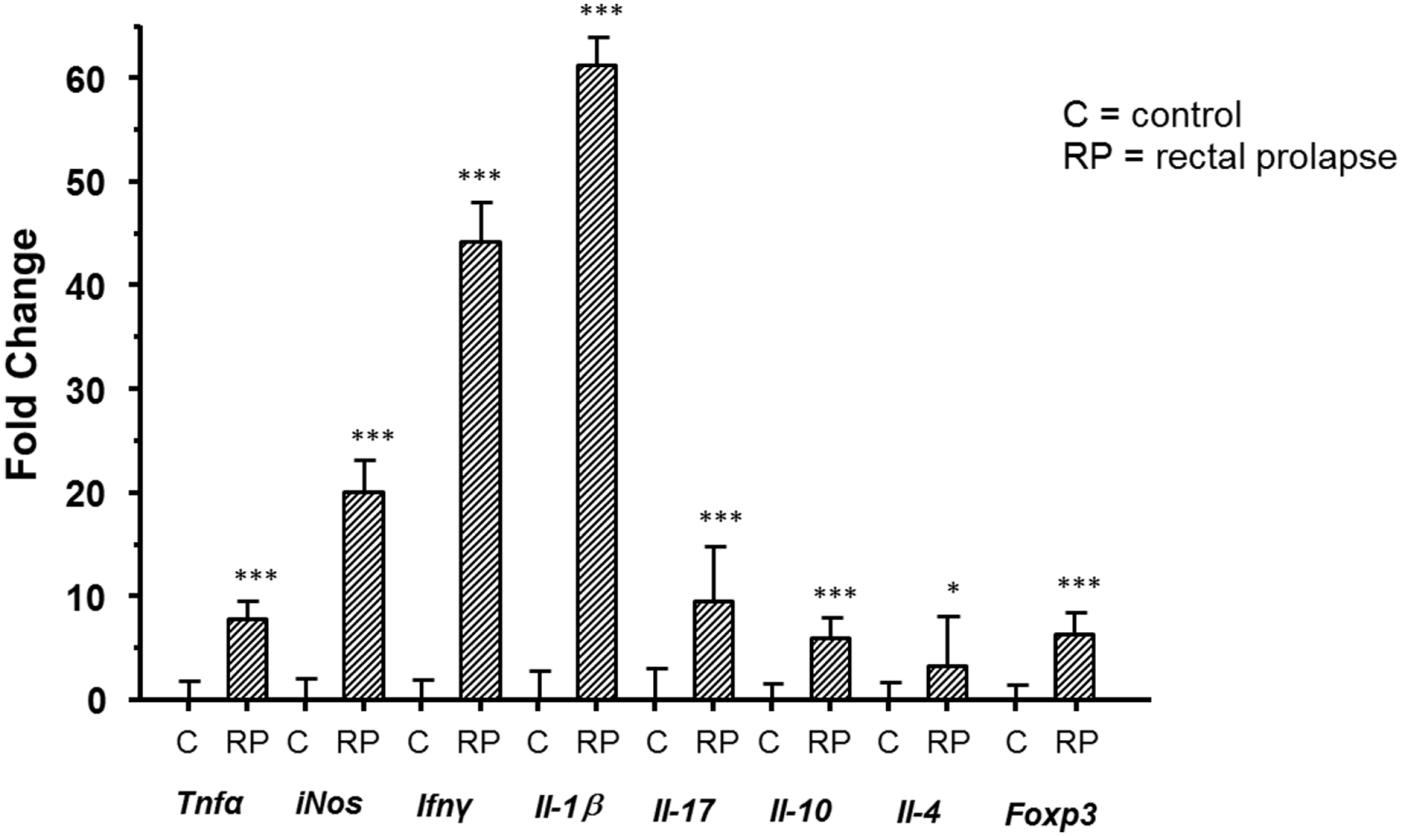
Lpd^-/-^ mice with RP produced significantly higher mRNA levels of rectal inflammatory mediators compared healthy controls. Total RNA prepared from rectal tissues of mice were evaluated by qPCR for expression levels of mRNA for select cytokines, which then were normalized to the expression of the house-keeping gene Gapdh. The Y axis represents the mean fold change (± standard deviation) of the mRNA levels in reference healthy controls. P values: *** <0.0001, * <0.026.

mRNA expression of inflammatory mediators was compared between rectal tissues of RP and EHS- mice. Expression of all markers was significantly elevated in the RP mice, with the pro-inflammatory factors including Ifn-γ, Tnf-α, iNOS, Il-1β, and Il-17, elevated at greater fold-change relative to the anti-inflammatory factors including Il-10, Il-4, and Foxp3 (Fig 6). We also compared expression in the proximal colons and rectums of RP mice, finding up-regulation in the rectum in all pro-inflammatory mediators measured, as well as a highly significant increase in IL-10 mRNA expression.

### Measurement of tissue infiltrating myeloid cells

**Fig 7:**
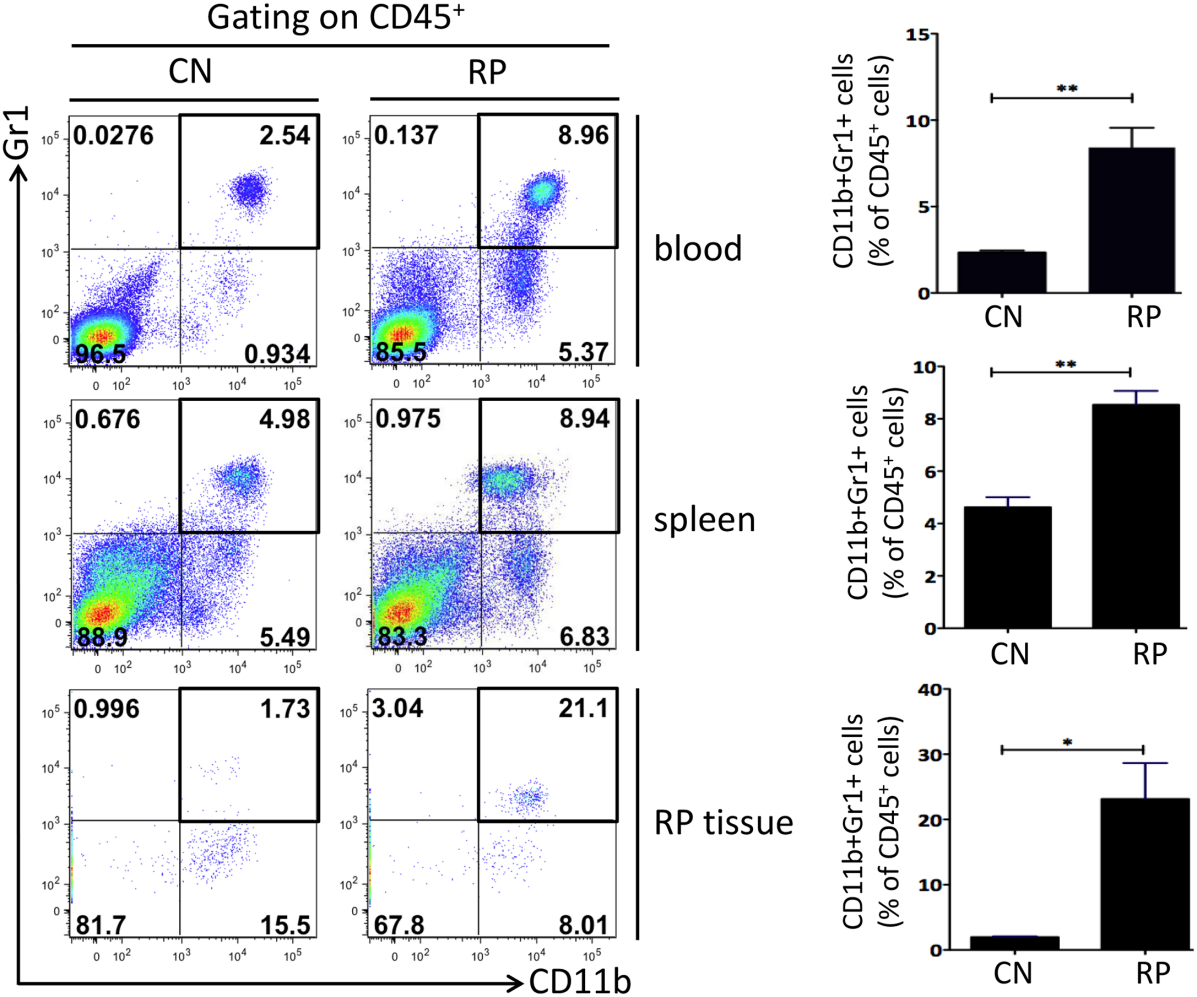
Flow analysis of CD45^+^CD11b^+^Gr1^+^ myeloid cells. Proportions of myeloid cells increased significantly in LPD^-/-^ RP mice blood, spleen, and rectal prolapse tissue. Left panel, representative flow plots of CD11b^+^Gr1^+^ myeloid cells from various tissue samples compared RP and control mice, gating on CD45^+^. Right panel, quantification of CD11b^+^Gr1^+^ myeloid cells in CD45^+^ population (n = 3). Error bars, mean ± s.e.m. P values were derived from an unpaired, two-tailed Student’s t-test (* P<0.05; ** P<0.01). CN = Clinically Normal; RP = Rectal Prolapse.

Although no change was seen when comparing prolapsed animals and clinically normal controls in the overall percentage of bone marrow myeloid cells (data not shown), we found a marked increase in both circulating and splenic CD45^+^CD11b^+^Gr1^+^ cells in the RP animals. This suggests that mobilization of inflammatory myeloid cells is one of the major events potentially contributing to RP formation in Lpd^-/-^ mice. We further evaluated the proportions of myeloid cells present in the prolapsed tissue. Interestingly, there was a strong infiltration in the RP tissue (>10 fold increase compared to control mice rectal tissue) of CD45^+^CD11b^+^Gr1^+^ myeloid cells (Fig 7).

## Discussion

Rectal prolapse is routinely observed in experimental mice under different settings that are highly influenced by the genotype, extent of intestinal inflammation, underlying neoplastic process or exposure to intestinal toxic insults such as DSS/AOM treatments [53]. While the occurrence of RP in *Helicobacter* spp. endemic mouse colonies is considered common in susceptible immunocompromised strains of mice, we have identified prolapse lesions in Lpd^-/-^ mice characterized histologically as rectal carcinoma associated with EHS-induced chronic inflammation. *Helicobacter* spp. infected LPD^-/-^ mice with RP differ from other mice with RP in that inflammatory lesions are localized to the rectal tissue with little or no inflammation in the cecum or colonic tissue proximal to the rectum. In contrast, immunocompromised mice that exhibit RP typically have a florid typhlocolitis. Diagnosis of rectal carcinoma in prolapsed rectal tissue can be difficult, as the exposure of mucosa can result in inflammatory changes that may mimic a more invasive appearance. Recommendations on evaluating mouse intestinal pathology have been developed to establish consistent criteria for distinguishing herniation from carcinoma [50]. Features of carcinoma in the epithelium include irregular/angulated glands, loss of mucosal lining in the invasive glands, mucus lakes, desmoplasia, and high-grade dysplasia. The RP lesions of Lpd mice fit into the former category, with 11 of 19 (58%) classified as invasive carcinoma. Consistent pathology among multiple mice of the same genotype, as observed in this study, is also highly suggestive of a true rectal inflammation and associated dysplastic progression leading to carcinoma rather than simple herniation.

Given the focal nature of the lesions, we interpret the initial insult to have originated at the very distal rectal glandular epithelium. This implies lamellipodin has a unique role for tissue integrity at the distal rectum and lack of lamellipodin predisposes this tissue to pathogenic properties of EHS infection. At this site, the epithelial cells transition from the mucosal simple columnar cells to keratinized stratified squamous cells of the anal region. This transitional zone is especially prone to tumorigenesis. Authors have reported that the anorectal zone in mice is populated by a unique population of stem cells, which they argue have extensive capabilities to proliferate, implicating these cells in tumorigenesis [54]. Transitional epithelia are common sites of neoplasia; examples include bladder cancer in dogs (transitional cell carcinoma occurring at the trigone) [55], cervical cancer in women (exclusively at the vaginal-cervical junction)[56], and distal esophageal cancer [57].

Despite decreasing levels of *Helicobacter* spp. being noted in the distal colon and rectum, detectable levels by qPCR were noted in the prolapsed tissue and also consistently demonstrated by FISH within the rectal lesions. We attribute this decline in bacterial load to reflect the normal colonization niche of *Helicobacter* spp. being the colon and cecum. In addition, it is possible that as the mucosal architecture becomes distorted with advancing disease, the rectal environment becomes less hospitable for EHS colonization. This phenomenon is seen in *H*. *pylori*-induced gastric adenocarcinoma in which organisms can no longer be detected in advanced stages of disease [58].

A highly significant pro-inflammatory response was observed in the prolapsed rectal tissue, far exceeding elevations in anti-inflammatory cytokines. Of note, Il-1β was particularly high at an over 60-fold increase in expression compared to that in EHS- control rectal tissue. Il-1β plays a pivotal proinflammatory role in *H*. *pylori*-induced gastric inflammation, DNA methylation, and gastric cancer [59, 60]. In a model of *H*. *felis*-associated gastritis and gastric cancer, infected transgenic mice that constitutively over-express IL-1β in parietal cells were shown to develop more severe lesions than infected WT control mice [59]. Increased IL-1β was accompanied by a rise in other pro-inflammatory cytokines (Tnf-α, Il-6, and Sdf-1α) in both the serum and stomach [59]. Il-1β also recruited myeloid-derived suppressor cells (MDSC), found to be elevated in the serum, spleen, and stomach; this occurred via an NF-кB signaling pathway [59]. The Il-1β mobilization of MDSCs was also proposed as an early mediator of neoplastic transformation in gastric tissue [59]. Evidence for a role for MDSCs includes the immune phenotyping data, comparing serum, spleens, and rectal tissue of clinically normal vs prolapsed tissue. We were able to demonstrate that CD11b^+^ Gr-1^+^ MDSC populations that were significantly elevated in all three of these locations in the mice with RP compared to normal mice. This population of myeloid cells has previously been shown to inhibit antigen-specific T-cell activation in the tumor microenvironment [61, 62]; and has been specifically associated with acceleration of both gastric and colonic carcinogenesis in Il-8Tg mice [63]. Localized increased numbers of these cells suggest an attempt at suppression of the many pro-inflammatory cytokines found in this same area. It also presents the possibility of immune dysfunction in these mice, previously unknown to have a defined immune phenotype.

It is plausible that, as with *H*. *pylori*- and *H*. *felis*-induced disease in the stomach, Il-1 β is mediating an EHS-induced inflammatory-associated neoplastic transformation in Lpd mice. Recently, Il-1β was shown to stimulate COX-2 production in colorectal fibroblasts, thereby enhancing proliferation and invasiveness of epithelial cancer cells [64]. Though COX-2 expression was not measured in our experiments, it has been shown to be overexpressed in colorectal cancers in humans and may be useful in assessing the inflammatory response in future investigations with this model [65]. Other mouse models of *H*. *hepaticus*-induced IBD have shown elevations in IL-1β correlating with severity of histologic lesions, as well as promoting a Th-17 type response and recruiting innate lymphoid cells and granulocytes [66]. In an mdr1a^-/-^ mouse model of IBD, infection with *Helicobacter* spp. was shown to be required for elevation of IL-1β mRNA levels; this was associated with a high-grade dysplastic response [67].

A statistically elevated number of H2AX positive epithelial cells in rectal carcinoma tissue is a biomarker for DNA damage induced by DNA double-strand breaks. Chronic *H*. *pylori* infection, a risk factor for gastric cancer, also elicits an increased expansion of H2AX, [68, 69]. Similarly, *H*. *hepaticus* induces lower bowel carcinoma in 129-Rag^-/-^ mice [37, 70]. Also noteworthy is the presence of cytolethal distending toxin in *H*. *hepaticus*; this toxin is responsible for inducing DNA double-strand damage breaks [71]. The presence of statistically elevated expression of H2AX in mice with rectal carcinoma also provides support for the EHS, or other bacteria, in driving a carcinogenic process.

Given the frequency with which *Helicobacter species* are linked to lower bowel inflammation in mice, the established use of *Helicobacter*-induced mouse models of IBD, and the abrogation of the RP phenotype with embryo transfer rederivation, we propose that EHS serve as an initiator of proctitis with subsequent development of neoplasia. Unfortunately, we are unable to monitor the course of pathogenesis with spontaneous disease; however, in our assessment of infected mice without RP, we noted sporadic cases of mild lower bowel inflammation. One 3 month old homozygous null male had mild proctitis, epithelial hyperplasia, and glandular degeneration. With the assumption that there is a period of chronic proctitis preceding prolapse, perhaps this young mouse with proctitis would have developed a prolapse at an older age. Although we observed a wide age range of mice with RP (4 to 13 months), we predict that with the controlled dose and time of inoculation in experimental rather than spontaneous infections, we may be able to more reliably predict a time course of disease. We must also acknowledge that through the rederivation process, these mice likely have GI flora changes in addition to the elimination of EHS. For example, in a recent study employing IL10^-/-^ C57BL mice infected with the same *H*. *hepaticus* strain 3B1, but located at 2 different institutions, mice in one location developed lesions of IBD while those maintained in the second location did not [72]. Microbiome analysis depicted different bacterial species in mice at the different sites, which may have, in part, influenced the susceptibility of the *H*. *hepaticus* infected mice to develop IBD [72]. Experimental infection with EHS, along with microbiome assessment of the lower bowel, will help to verify whether there is a definite EHS association with rectal carcinoma in Lpd^-/-^ mice.

Many viral and bacterial pathogens, both extracellular and intracellular, are known to use host cell membrane and cytoskeletal components to induce their respective diseases, with actin dynamics being a frequent target [73]. Lpd is known to co-localize to actin pedestals formed by enteropathogenic *E*. *coli* (EPEC) at sites of attachment to host cells [9]. In this process, the bacterial intimin receptor, Tir, is translocated into the host cell to activate the SHIP-2 phosphatase; SHIP-2 then recruits Lpd to induce actin pedestal formation [74]. Thus, while EPEC can manipulate Lpd to inflict cell damage, *Helicobacter*-associated disease in the absence of Lpd suggests Lpd plays a role in host defense, specifically at the very distal rectum. Unfortunately, the relevance of Lpd to normal gastrointestinal epithelial function is unknown. Given the pivotal role of Lpd in cell motility and membrane projections, its absence may simply weaken epithelial integrity of rectal tissue. As evidence of lamellipodin’s key role in cell stability and migration within the extracellular matrix, it has shown to be responsive to Rac signaling by localizing actin to the peripheral cytoskeleton via the Arp2/3 complex [15, 75], contributing to intracellular stiffening and mechanotransduction in MEFs [75]. Adaptor proteins in the Cas family within this FAK-Cas-Rac-Lpd signaling cascade also have oncogenic potential. Mutations causing phosphorylation in p130CAS have been identified in colon cancers sensitive to the kinase inhibitor Dasatinib [76]. CAS-L is shown to be upregulated in colorectal tumors in response to Wnt signaling, correlating with cell invasion and migration [77]. Further examination of identified oncogenic components of these pathways and their relationship to Lpd may help to further define Lpd’s role in colorectal carcinogenesis.

However, Lpd could have a more significant adaptive role. We recently reported that WASP-deficient mice infected with *H*. *bilis* develop colitis and colitis-associated cancer. WASP (Wiskott-Aldrich syndrome protein) is a hematopoietic—specific marker that mediates T-cell receptor-dependent signaling, as well as phagocytosis and podosome formation [78, 79]. Interestingly, the WASP family of proteins is also involved in intracellular signaling and actin polymerization via the Arp2/3 pathway [78]. WASP and the Ena/VASP family (including Lpd) have been shown to co-localize upon activation of the Fcγ receptor, subsequently initiating actin reorganization for phagosome formation in macrophages [80]. Thus, conceivably the loss of Lpd may impair phagocytosis in the rectum, making the epithelium more vulnerable to pathogenic bacteria.

Our understanding of EHS virulence factors is mostly based on studies of *H*. *hepaticus* [81]. Although approximately half of Lpd mice with RP were infected with *H*. *hepaticus*, all mice were infected with *H*. *typhlonius*, suggesting that *H*. *typhlonius* plays a major role in eliciting rectal inflammation in Lpd-/- mice. In general, EHS are known to be mucosal-associated organisms that remain extracellular, but perturb host cells via various virulence factors including cytolethal distending toxin, and type VI secretion systems which have been identified in *H*. *hepaticus* strains [81]. Studies on the gastric pathogen *H*. *pylori* have shown vasodilator-stimulated phosphoprotein (VASP) to be a target protein of this *Helicobacter* species, with VASP mediating elongation of gastric adenocarcinoma cells [82]. In this study, the virulence factor CagA- was shown to be required for protein kinase G-driven phosphorylation of VASP, another Ena-VASP protein that, like lamellipodin, regulates actin formations and cell motility. While CagA is a *H*. *pylori*-specific virulence factor, it illustrates the ability of this genus of bacteria to manipulate Ena-VASP proteins.

Other possible mechanisms of RP could include connective tissue disorders, particularly given lamellipodin’s role in fibroblast migration. The distal bowel of the mouse, which is relatively short and lacks a significant serosal rigidity, may be predisposed to loss of mechanical stability due to abnormal cytoskeletal dynamics. Likewise, because Lpd is critical in axon migration and function, there may be an unrecognized anomaly in enteric innervation that impairs lower bowel motility, similar to Hirschprung’s disease. We have not observed clinical tenesmus or megacolon that would suggest this defect in Lpd^-/-^ mice. Other systemic signs might also be expected with generalized nervous or connective tissue abnormalities. Mouse models of pelvic floor dysfunction are female-predominant phenotypes and more often involve urinary incontinence and uterine or bladder prolapse rather than rectal prolapse [83, 84]. Loss of elastin-associated proteins such as lysyl oxidase like-1 and fibulin-5, are generally responsible for these phenotypes [83, 84]. Rectal prolapse spontaneously occurs in mice that overexpress hepatocyte growth factor/scatter factor, causing chronic enteritis with pseudo-obstruction [85]. While lesions are more severe distally, this phenotype is present in both *Helicobacter* spp- infected and with *Helicobacter spp*.-free mice, and lesions were not neoplastic [85].

The sex differences observed in this model are also striking, with 89% of cases being male. This is in agreement with rectal tumor statistics in humans, which are more common in males [86]. Variants in estrogen synthase are linked to risk of colorectal cancers, with different polymorphisms associated with colon versus rectal cancers; these may modulate inflammatory and oncogenic pathways [87]. In humans, estrogens have been shown to be protective against both colorectal cancer [88] and gastric cancer [89]. This protective phenomenon is translatable to the INS/GAS mouse model of *H*. *pylori*-associated gastric cancers, where males are more prone to develop gastric cancer [90, 91]. Likewise, Lpd-deficient mice may be useful in studying estrogen modulation of inflammatory and oncogenic pathways in EHS-associated rectal carcinoma.

We are unaware of any established spontaneous mouse models of rectal carcinoma. Distinction of rectal tumors as a subset of colon cancer is difficult, as they are often grouped collectively as colorectal cancer. These tumors rank as the third most common neoplasm in the US (and the most common gastrointestinal neoplasm). The most recent CDC report on cancer incidence shows colorectal tumors make up over half of all gastrointestinal tumors; of these, a significant proportion (28%), are within the rectum and rectosigmoid junction [86].

This investigation has provided an EHS association with rectal cancer in Lpd^-/-^ mice, particularly in males. Further experiments will entail experimental infection with EHS to establish causality of rectal carcinoma in this mouse model. If successful, this new mouse model may also elicit mechanisms of EHS pathogenesis, particularly the manipulation of actin-dynamics and estrogen-modulated inflammation.
